# Exploring Different Effects of Exclusive Enteral Nutrition (EEN) and Corticosteroids on the Gut Microbiome in Crohn's Disease Based on a Three-Stage Strategy

**DOI:** 10.1155/2022/6147124

**Published:** 2022-07-27

**Authors:** Dong Guo, Liang Fang, Ruiqing Liu, Yu Li, Liang Lv, Zhaojiao Niu, Dong Chen, Yanbing Zhou, Weiming Zhu

**Affiliations:** ^1^Department of Gastrointestinal Surgery, Affiliated Hospital of Qingdao University, No. 16 Jiangsu Road, Qingdao, Shandong, China; ^2^Department of Gastroenterology, The affiliated hospital of Qingdao University, Qingdao, Shandong, China; ^3^Department of General Surgery, Jinling Hospital, Medical School of Nanjing University, Nanjing, Jiangsu Province, China

## Abstract

The objective of this study was to compare the efficacy of exclusive enteral nutrition (EEN) and corticosteroids on the gut microbiome in Crohn's disease. *Methods*. Data were collected for 16 patients newly diagnosed with CD as the test group and 10 healthy volunteers as the control group. The 16 patients were randomly divided into the EEN group and the corticosteroids group. For subsequent analysis, 6 patients in the EEN group with follow-up were enrolled to compare the 0-month, 1-month, and 3-month outcomes. We analyzed and compared gut microbiota between different groups in 3 stages. To evaluate the clinical outcome of treatment, erythrocyte sedimentation rate (ESR), C-reactive protein (CRP), hemoglobin (HB), albumin (ALB), and Crohn's disease activity (CDAI) were recorded. *Results*. There are significant differences in microbiota between patients with CD and healthy people, and there are intuitive differences in the main components of the microbiota. 16 patients were included in stage 2, in which both corticosteroids and EEN can induce CD remission well. However, corticosteroids have a greater impact on inflammatory indicators, while EEN has a more obvious effect on nutritional indicators. Principal component analysis suggests that there are different compositional changes in the gut microbiome after corticosteroids and EEN treatment. After 3 months of dynamic observation, we found that EEN can effectively maintain CD remission, reduce inflammatory indicators, and improve nutritional indicators. *Conclusions*. Both EEN and corticosteroids can increase the diversity of the microbiome in inducing CD remission, while they have different effects on the proportion of microbiome species. This trial is registered with NCT02056418.

## 1. Introduction

Crohn's disease (CD) is a transmural, chronic, nonspecific inflammatory disorder affecting the entire digestive tract. In recent years, the incidence of CD is increasing in China, which could increase the morbidity associated with the disease in patients with CD. Some research ascribed the development of CD to the interaction of patients' genetics factors, epigenetic factors, and microbial exposure [1–4]. However, the specific etiology of CD is still not clear.

EEN is usually regarded as a first-line treatment for CD, which could lead to a higher rate of mucosal healing. Compared with corticosteroid treatment, EEN could avoid some short-term and long-term side effects. Moreover, EEN could increase the likelihood of absorption and the following growth.

Therefore, in this study, we compared the outcome of induction therapy with exclusive enteral nutrition (EEN) to that of corticosteroids and to further compare the gut microbial composition between the two induction therapy strategies.

The microbial composition plays an important role in the pathogenesis of CD. Recent research showed in children's CD, EEN can affect the microbiota while inducing remission [4]. There is still a lot of controversy about what changed after EEN treatment [5]. Corticosteroids are still the main intervention commonly used to induce the remission of active CD, of which long-term use will bring a lot of side effects. Generally speaking, they cannot improve the prognosis of CD and cannot promote mucosal healing [2]. In a recent study, they compared mucosal healing and bacterial composition in response to enteral nutrition with steroid-based induction therapy. They found both steroid and EEN-induced clinical remission. However, it demonstrated patients with EEN-induced remission showed a higher rate of mucosal healing and this was associated with a different gut microbiota compositional shift in these children [6].

In this study, we took a three-stage strategy: compared patients with CD with healthy people, compared EEN with corticosteroids treatment, and compared the follow-up in different times. The study compared the effect of 2 treatments on patients with CD and has a cohort of healthy controls to compare against, in which patients on EEN were sampled longitudinally. The results indicate that both corticosteroids and EEN have a good effect of inducing remission. Corticosteroids have a significant effect on improving inflammation indicators, while EEN has a relatively significant improvement on nutritional indicators. Both treatment methods have a tendency to increase the diversity of the intestinal tract, yet completely different effects on the species and composition of the microbiota or on specific bacterial species. Corticosteroids have a greater impact on the microbiota. Our study demonstrated that the process of EEN-induced CD remission can change the proportion of microbiota, increase the diversity of the microbiota, and make it tend to be more similar to that seen in healthy individuals with therapy. This may be the mechanism of EEN-induced CD remission and further experiments are needed.

## 2. Material and Methods

### 2.1. Study Workflow

**Figure 1:**
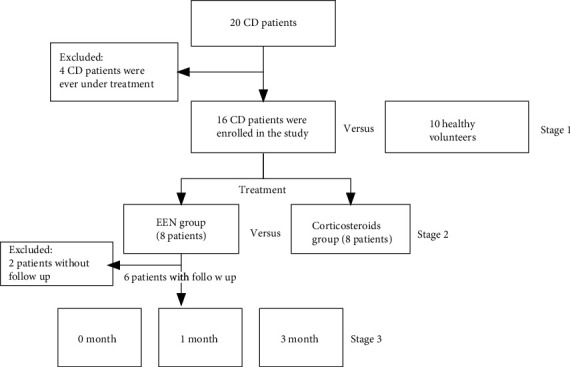
The flow chart of the three-stage research.

To better understand the effects of exclusive enteral nutrition (EEN) in patients with Crohn's disease and to compare with the effects of corticosteroids, we took a three-stage strategy: Patients with CD verse health volunteers, EEN treatment verse corticosteroids, and inner comparison 0, 1, and 3 months after EEN treatment (Figure 1).

### 2.2. Patients Selection and Exclusion Criteria

This study was approved by the Medical Ethics Committee of Nanjing General Hospital of Nanjing Military Command. From August 2013 to September 2014, inpatient volunteers were treated at the General Surgery Inflammatory Bowel Disease Center of Nanjing General Hospital of Nanjing Military Command. Patients need to meet all the selection criteria (satisfy all the selection criteria and not violate any exclusion criteria).

Patients that were 18–75 years old with CD, diagnosed through histopathology from endoscopic tissue biopsies, serum bloodwork (C-reactive protein (CRP) ≥10 mg/L), abdominal CT, and magnetic resonance enterography, were approached for recruitment. Patients were excluded if they were older than 75 years or younger than 18 years old, ever received any treatment. Patients were also excluded if they were in a very severe condition, including CDAI> =450, patients with extraintestinal fistula, abdominal abscess, or short bowel syndrome, and patients with other severe liver, kidney, cardiopulmonary, and nervous system diseases and immune-related diseases.

The healthy controls were subjects of 18-75 years old, without systemic diseases, without drinking and smoking history, and without a recent medication history.

### 2.3. Intervention

Patients in the EEN group were given enteral nutrition to induce remission. The specific scheme was as follows: the selected patients were given a nasal feeding tube and total enteral nutrition for 4 weeks. In the course of treatment, people can drink water, forbid other meals, forbid other drugs, and, if necessary, take pheneperidine orally to treat diarrhea. Heat card refers to indirect energy measurement results or 25-30 kcal/kg/d. Patients were hospitalized for treatment according to their condition or received family enteral nutrition treatment under the guidance of doctors. During the treatment, doctors and nurses should closely observe, control the infusion speed, adjust the temperature, prevent aspiration, and try to reduce patients' enteral nutrition intolerance and complications. If the patient has enteral nutrition intolerance or complications, it should be adjusted in time according to the specific situation and treated symptomatically. If the patient does not tolerate the nasal feeding tube, adjust and replace the nasal feeding tube in time, or change to oral-nasal feeding alternately.

The patients in the steroid group were orally or intravenously administered with a dose equivalent to 0.75~1 mg • kg^−1^ • d^−1^ prednisone. After symptom relief, 5 mg was reduced every week, and when it was reduced to 20 mg/d, 2.5 mg was reduced every week until it was stopped. During the treatment, the patient ate normally and was forbidden to take other drugs. Pay attention to drug-related adverse reactions and handle them accordingly. Calcium and vitamin D should be supplemented at the same time. After adjusting the symptomatic treatment, if the hormone is intolerant, or the complications are not controlled, or the induction fails and the condition intensifies, the treatment plan shall be changed in time.

### 2.4. 16S rRNA Gene Sequencing

In order to get the gut microbiota diversity, we collected fecal samples of the participants for 16S rRNA gene sequencing. At the time of baseline collection, patients have not been treated yet. Within 3 days after enrollment, fecal samples were collected. The workflow of sample collection is as follows. In a clean environment, subjects were asked to use sterile cups to collect fecal samples. The experimenter collected fecal samples at multiple points in the super clean workbench, sub package to liquid nitrogen cryopreservation tube (2 parts, 2 ml each). After numbering, place it in liquid nitrogen for quick freezing. The above operations shall not exceed 30 minutes. After overnight, it shall be placed in the -80°C deep low-temperature refrigerator for storage. After samples collection, fresh samples were stored at -80°C refrigerators or in liquid nitrogen tanks within 6 hours. The genomic DNA of bacteria was extracted from samples with the freeze–thaw method. The 16S rDNA was PCR-amplified and then sequenced on the MiSeq PE250 system (Illumina, USA). The primer sequence was F: 5′GTGCCAGCMGCCGCGGTAA3′ and R:5′GGACTACHVGGGTWTCTAAT3′.

### 2.5. Operational Taxonomic Unit (OTU) Clustering

OTU and abundance analysis includes preliminary OTU statistics, Venn diagram analysis, and PCA, which can provide a preliminary understanding of the species abundance and main component composition of the samples. The analysis of species and their abundance can obtain the species composition ratio of each sample at each taxonomic level (phyla, class, order, family, genera, and species), reflecting the community structure of the sample at different taxonomic levels. The detailed OTU count of each sample was provided in the supplementary table (available here).

### 2.6. Statistical Analysis

Continuous variables were expressed in the form of mean ± SD or mean ± SE. A *t* test/nonparametric test was used for comparison between groups. *p* < 0.05 is a statistically significant difference. The difference in the abundance of microbial communities was analyzed and the FDR (false discovery rate) was used to evaluate the significance of the difference. We used SPSS 18.0 and GraphPad Prism 5 for statistical analysis and graphing. For bioinformatics analysis, Qiime 1.7.0 was used to cluster sequencing data to form OTU, and annotate according to the database to make a dilution curve. Use R software to make Venn diagram and principal component analysis diagram. Combining the database Qiime to generate species abundance tables and multisample species distribution maps at different taxonomic levels (phyla, class, order, family, genera, and species). The specific values used the metastats command in the software mothur and the R software (rank-sum test, Fisher's exact test, chi-square test, *t* test, square difference test) Perform significant difference analysis between groups, and the *p*-value correction method was “BH” method.

## 3. Results

A total of 20 patients were involved in the study. After signing the informed consent form, patients with CD were randomly divided into a corticosteroid group and an enteral nutrition group (10 patients in each group). They received corticosteroid therapy and EEN, respectively, to induce remission. Among them, 4 patients were excluded (ever were under treatment before), and a total of 16 patients completed the study (Table 1).

### 3.1. Clinical Outcome

In stage 2, Table 2 shows that after 4 weeks of corticosteroids therapy, CDAI decreased significantly, and the inflammatory indicators CRP and ESR also decreased significantly after induction. Nutritional indicators that include HB (hemoglobin), ALB (albumin), body weight, and SM (skeletal muscle) were significantly improved, and the quality of HB was significantly improved before and after (102.45 ± 14.23 vs. 117.50 ± 10.85, *p* =0.032). After 4 weeks of EEN treatment, CDAI decreased significantly, and inflammatory indicators (CRP and ESR) were also significantly improved. Nutritional indicators improved; HB and ALB were significantly improved (108.87 ± 12.85 vs 128.36 ± 9.91 *p* =0.004; 30.86 ± 3.05 vs. 37.94 ± 3.40 *p* =0.001); body weight and SM increased, but not statistically significant (Table 2). Due to the small number of research subjects, the induction effect cannot be evaluated. We found that corticosteroids have a greater impact on inflammation indicators, while EEN has a more obvious effect on nutritional indicators. It is worth pointing out that EEN is significantly better than corticosteroids therapy after induction. The patient's serum albumin level was significantly higher than that of corticosteroid therapy (37.94 ± 3.40 vs. 33.19 ± 2.36, *p* =0.006). All in all, corticosteroids and EEN have good clinical effects on the research population.

In stage 3, Table 3 shows that patients who have completed the 3-month follow-up have achieved good clinical results when EEN maintains remission, and their clinical symptoms are well controlled. CDAI indicates disease remission, and there is a downward trend. Inflammatory indicators (erythrocyte sedimentation rate and C-reactive protein) also showed a downward trend when they were maintained in remission by EEN. Nutritional indicators, including hemoglobin, albumin, body weight, and skeletal muscle, steadily increase during the maintenance of remission of EEN. The results suggest that when EEN maintains CD remission, it can well control disease activity and effectively maintain disease remission, reduce inflammation indicators, and improve nutritional status.

### 3.2. PCA

Through PCA, we intuitively observed the changes in the composition of the microbiota in different stages. On the two-dimensional coordinate graph, its coordinate axis takes two characteristic values that can reflect the maximum variance value. If the two samples are closer together, the composition of the microbiota of the two samples is similar.

**Figure 2:**
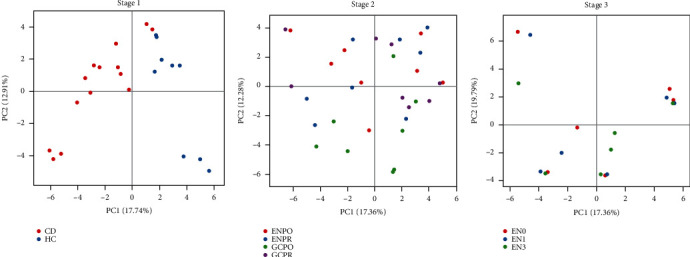
PCA in three stage. Abbreviations: HC: health control; CD: Crohn's Disease; GCPR: before corticosteroids treatment; ENPR: before EEN treatment; GCPO: after corticosteroids treatment; ENPO: after EEN treatment; EN0: 0 month after EEN treatment; EN1: 1 month after EEN treatment; EN3: 3 months after EEN treatment.

In stage 1, the health group and CD group are obviously distinct, indicating that the microbiota is different in composition between the two groups (Figure 2).

In stage 2, purple and blue, respectively, represent samples before corticosteroids treatment (GCPR) and before EEN treatment (ENPR). There is no significant trend of aggregation and separation between samples, and the distribution is even (*p* >0.05). The green dot represents the sample after corticosteroids treatment (GCPO), and the red represents the sample after EEN treatment (ENPO). It can be observed that the two groups are separated in the direction of PC2, indicating that the microbiota is different after corticosteroids and EEN treatment changes in composition.

In stage 3, red, blue and green, respectively, represent samples 0-month, 1-month, and 3-month follow-up. In this part, some 3-color clusters appear (labeled by the red circles), of which one cluster represents one sample, illustrating that the maintaining therapy of EEN has little effect on microbiota.

### 3.3. Taxonomic Abundance Analysis

**Figure 3:**
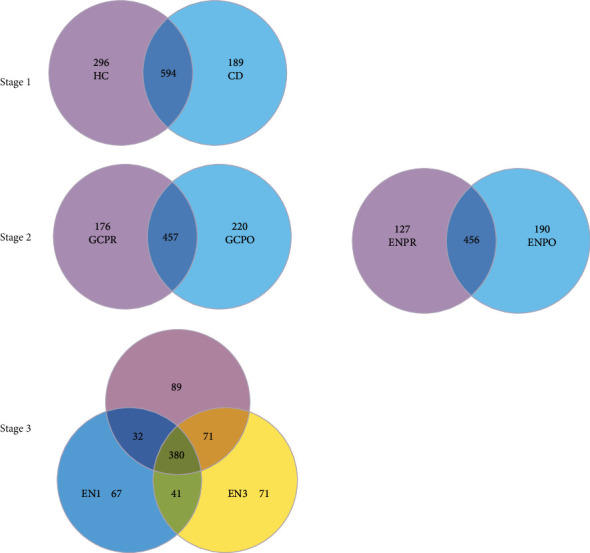
The number of OTU in different groups. Abbreviations: HC: health control; CD: Crohn's Disease; GCPR: before corticosteroids treatment; ENPR: before EEN treatment; GCPO: after corticosteroids treatment; ENPO: after EEN treatment; EN0: 0 month after EEN treatment; EN1: 1 month after EEN treatment; EN3: 3 months after EEN treatment.

In stage 1, the number of OTU in the health group is significantly higher than in the CD group (241.6 ± 75.5 vs. 382.3 ± 51.4, *p* <0.001). Venn diagram shows that compared with the health group, the CD group has a smaller abundance (890 vs.783). The health group and CD group have an intersection of 594 OUT (Figure 3).

In stage 2, we compare the abundance of OTU before and after treatment. Results showed that in the corticosteroids group after treatment, 176 OTU disappeared and 220 new OTU were added, and in the EEN group after treatment, 127 OTU disappeared and 190 new OTU were added, which illustrated that corticosteroids had a more profound effect on the richness of microbes of patients than EEN treatment.

In stage 3, we want to make clear the long-term effect of EEN treatment on patients with CD.

In the EEN group, after 3-month follow-up, 121 OTU disappeared and 112 new OTU added, which are more than 1-month follow-up.

### 3.4. Taxonomic Abundance Analysis

**Figure 4:**
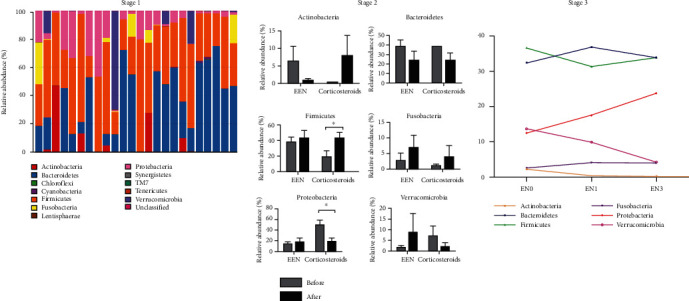
Phyla measurement of the 3 stage. Abbreviations: EN0: 0 month after EEN treatment; EN1: 1 month after EEN treatment; EN3: 3 months after EEN treatment.

A total of 12 phyla were measured, among which we selected the main phyla (relative abundance of the two groups>1%), observed, and compared the effects of different groups on the microbiota (Figure 4).

In stage 1, in the CD group, the abundance of Lentisphaerae, Synergistetes, and Chloroflexi was extremely low or even not detected. Compared with the healthy control group, the CD group had a significant increase in fecal Proteobacteria (16.3 ± 3.90 vs. 3.53 ± 0.99, *p* = 0.007, FDR = 0.0216); and Cyanobacteria (0.14 ± 0.01 × 10-2) vs. 0.38 ± 0.20, *p* = 0.009, FDR = 0.0221), Bacteroidetes (22.04 ± 6.48 vs. 50.3 ± 5.73, *p* = 0.01, FDR = 0.0276), and Tenericutes (0.10 ± 0.10 × 10-3 vs. 2.50) ± 1.08 × 10-2, *p* =0.001, FDR = 0.043) significantly reduced.

In stage 2, the abundance of Actinobacteria and Bacteroidetes decreased after EEN treatment, but increased after corticosteroids treatment (*p* < 0.05). The abundance of Firmicutes and Fusobacteria showed an upward trend after EEN and corticosteroids treatment (*p* < 0.05). The abundance of Proteobacteria and Verrucomicrobia increased after EEN treatment but decreased after corticosteroids treatment (*p* < 0.05). These findings suggest that after EEN and corticosteroids induce CD remission, both have a profound impact on the microbiota, but they are microbiota-specific.

In stage 3, Synergistetes appeared after EEN maintained remission for 3 months; Tenericutes and Cyanobacteria gradually became undetectable during EEN treatment. When selecting the main phyla (relative abundance>1%), and continuously observing the influence of EEN on the abundance of specific microbiota, there is no statistical difference in its changes, but there is a clear trend. The relative abundance of Fusobacteria and Proteobacteria in EEN maintained a rising trend during the remission process, while the relative abundance of Verrucomicrobia and Actinobacteria showed a downward trend. Bacteroidetes and Firmicutes maintained remission in EEN with different trends. The relative abundance of Bacteroidetes in EEN maintained remission for 1 month showed an increasing trend and continued to maintain treatment for 2 months, its abundance shows a downward trend, while Firmicutes is just the opposite.

## 4. Discussion

The pathogenesis of CD is not clear, which is related to multiple factors such as genetics, environment, and microbiota. Among them, the interaction between the microbiota and the occurrence and development of CD has become one of the main hotspots in exploring the pathogenesis of CD [7–9]. From clinical studies that described the microbiota and clinical manifestations to genetic studies based on the interaction between susceptibility genes and symbiotic bacteria, it has been confirmed that CD is closely related to microbiota [10, 11]. In the treatment of CD, EEN has received more attention. In addition to its ability to induce and maintain CD remission, EEN has the advantages of promoting mucosal healing, improving nutritional status, regulating mucosal microbiota, and improving quality of life. Meanwhile, it is favored by clinicians that many areas have become the first choice for the treatment of CD, especially children's CD [12–17]. At present, there is still no definite theory to explain how EEN induces CD remission. Scholars elucidate the mechanism from maintaining the intestinal mucosal barrier, regulating the microbiota, inducing mucosal healing, regulating microRNA, and reducing mesenteric fat [3, 4]. Studies have confirmed that the EEN treatment process can have an impact on the microbiota, including the proportion of the main microbiota, the “key” bacteria and the diversity of the microbiota [5]. At present, the research on EEN treatment and microbiota has not reached a conclusive conclusion. There is a similar study on the role of EEN in ulcerative colitis (UC), in which they demonstrated that the gut microbiota of pediatric UC and patients with CD was most influenced by patients' success or failure to achieve remission to make it largely independent of the choice of treatment or disease type [18].

In this study, we first tried a three-stage strategy to make clear the mechanism of how EEN and corticosteroids therapy influenced patients with CD. Significant differences were found in microbiota between patients with CD and healthy people, as well as intuitive differences in the main components of the microbiota. A total of 16 patients were included in 2 stage, in which both corticosteroids and EEN can induce CD remission well. At the same time, it was found that corticosteroids have a greater impact on inflammatory indicators, while EEN has a more obvious effect on nutritional indicators. After completing 4 weeks treatment, results suggest that corticosteroids and EEN have different effects on the microbiota. According to preliminary OTU statistical analysis, we found that corticosteroids therapy has a greater effect on the microbiota than EEN. Principal component analysis suggests that there are different compositional changes in the microbiota after corticosteroids and EEN treatment. There are also different effects on the composition of the microbiota, *Actinobacteria* and *Bacteroidetes*. Their abundance decreased after EEN treatment and increased after corticosteroids treatment. The abundances of *Firmicutes* and *Fusobacteria* showed an upward trend after EEN and corticosteroids treatment. The abundance of *Proteobacteria* and *Verrucomicrobia* increased after EEN treatment but decreased after corticosteroids treatment. The study could suggest that both EEN and corticosteroids therapy have a profound impact on the microbiota. Meanwhile, follow-up analysis showed that EEN can effectively maintain CD remission, reduce inflammatory indicators, and improve nutritional indicators.

Recent studies have confirmed that *Faecalibacterium prausnitzii* can reduce intestinal inflammation and play an anti-inflammatory effect. The reduction of its abundance can increase the recurrence of CD, which is greatly reduced in the intestinal body of patients with CD [19]. However, this study confirmed that it has an increasing trend after EEN treatment and decreases after corticosteroids treatment. The increase of CD *Escherichia* coli, especially the increase of mucoadhesive *Escherichia* coli, will promote intestinal inflammation and aggravate intestinal mucosal damage [20]. The abundance of EEN increased after induction of remission but decreased significantly after corticosteroids induction. The results prove that the composition of the microbiota is complex and interacts closely with the host. EEN and corticosteroids, as two different induction methods, have an impact on the composition of the intestinal bacteria. In the process of EEN and corticosteroids-induced CD remission, the diversity of the microbiota has an increasing trend. The effect of corticosteroids is greater, but there is no statistical difference. After EEN-induced remission, the components of *Fusobacteria* and *Proteobacteria* increased, and the *Actinomycota* gradually decreased, showing a trend toward healthy people.

## 5. Conclusion

In conclusion, there are significant differences in microbiota between patients with CD and healthy people.

Preliminary OTU statistical analysis shows that compared with EEN, corticosteroids therapy has a greater effect on the microbiota. Principal component analysis suggests that there are different compositional changes in the microbiota after hormone and EEN treatment. Both corticosteroids and EEN have a good effect of inducing remission.

After 3 months of dynamic observation, we found that EEN can effectively maintain CD remission, reduce inflammatory indicators, and improve nutritional indicators. Maintaining CD remission has less impact on the microbiota, which could increase the diversity of microbiota. The analysis showed that patients became more similar to healthy controls after the EEN treatment. This may be one of the mechanisms by which EEN induces and maintains CD remission.

Our work proved that both EEN and corticosteroids can increase the diversity of the microbiome in inducing CD remission, while they have different effects on the proportion of microbiome species. However, our work is at the macroscopic level and more experiments need to be conducted to explore the function of a specific microbiome species.

## Figures and Tables

**Table 1:** Baseline characteristics of the study population.

	Corticosteroids	EEN
N	8	8
Age at diagnosis	29.3	30.8
Males	4	5
Disease location		
Ileal (L1)	2	1
Colonic (L2)	2	2
Ileocolonic (L3)	4	5
Disease behavior		
B1	3	2
B2	5	6
History of corticosteroids	3	1
CDAI	274.81	253.86

Abbreviations: EEN: exclusive enteral nutrition; CDAI: Crohn's disease activity index.

**Table 2:** Clinical outcome of corticosteroids and EEN therapy.

	Subject	Before (*n* =8)	After (*n* =8)	*p* value
Corticosteroids (*n* =8)	CDAI	274.81 ± 92.14	98.63 ± 28.05	<0.001
	ES (mm/h)	37.70 ± 3.23	18.69 ± 3.88	<0.001
	CRP (mg/L)	35.79 ± 15.89	12.49 ± 3.80	0.001
	HB (g/L)	102.45 ± 14.23	117.50 ± 10.85	0.032
	ALB (mg/L)	31.00 ± 4.38	33.19 ± 2.36	0.234
	W (kg)	47.23 ± 10.21	48.14 ± 10.99	0.866
	SMM (kg)	21.68 ± 6.07	22.00 ± 6.19	0.920
EEN (*n* =8)	CDAI	253.86 ± 71.69	103.71 ± 44.13	<0.001
	ESR (mm/h)	35.94 ± 12.25	19.0 ± 4.69	0.003
	CRP (mg/L)	33.55 ± 14.28	13.88 ± 5.96	0.003
	HB (g/L)	108.87 ± 12.85	128.36 ± 9.91	0.004
	ALB (mg/L)	30.86 ± 3.05	37.94 ± 3.40	0.001
	W (kg)	47.81 ± 8.23	49.19 ± 7.69	0.735
	SMM (kg)	21.63 ± 5.61	23.01 ± 5.07	0.618

Abbreviations: CDAI: Crohn's disease activity index; ESR: erythrocyte sedimentation rate; CRP: C-reactive protein; HB: hemoglobin; ALB: albumin; SM: skeletal muscle; before: before treatment; after: after treatment; W: weight.

**Table 3:** Clinical outcome of EEN therapy in 0-month, 1-month, and 3-month follow-up.

	ENN0 (*n* =6)	ENN1 (*n* =6)	ENN3 (*n* =6)
CDAI	95.62 ± 18.39	94.83 ± 20.26	85.25 ± 16.32
ESR (mm/h)	17.67 ± 3.50	16.50 ± 3.27	16.83 ± 2.93
CRP (mg/L)	12.17 ± 3.19	9.67 ± 3.83	7.92 ± 3.01
HB (g/L)	128.07 ± 10.14	129.33 ± 12.88	132.42 ± 10.36
ALB (mg/L)	39.25 ± 2.52	39.33 ± 2.42	41.50 ± 2.26
W (kg)	46.75 ± 6.80	47.42 ± 6.34	49.08 ± 5.51
SMM (kg)	21.47 ± 4.67	21.73 ± 4.44	22.78 ± 4.33

Abbreviations: CDAI: Crohn's disease activity index; ESR: erythrocyte sedimentation rate; CRP: C-reactive protein; HB: hemoglobin; ALB: albumin; SM: skeletal muscle; W: weight.

## Data Availability

The data used to support the findings of this study are available from the corresponding author upon request.

## Supplementary Materials

Supplementary Materials A full OTU count table was provided in the supplementary table.
