# Supplementary material for: Exploring Different Effects of Exclusive Enteral Nutrition (EEN) and Corticosteroids on the Gut Microbiome in Crohn's Disease Based on a Three-Stage Strategy
Source: Gastroenterol Res Pract. 2022 Jul 27;2022:6147124. doi: 10.1155/2022/6147124 (PMC9348958; doi:10.1155/2022/6147124)
Supplement: Supplementary file 1 [file 6147124.f1.docx]

	1	2	3	4	5	6	7	8	9	
ENPO1	34.25482	16.30976	13.74401	10.82876	5.161894	3.196961	2.375219	2.174167	2.017534	1.367[illegible]
ENPO2	45.40524	14.89338	6.251186	6.007491	5.819725	3.531586	3.020225	1.348315	1.278402	1.152[illegible]
ENPO3	20.47135	17.09331	14.22212	13.54158	9.7032	3.271733	2.677563	2.105223	1.420883	1.375[illegible]
ENPO4	17.4688	13.11403	11.27464	7.259957	5.606783	3.982349	3.910497	3.733265	3.193184	2.763[illegible]
ENPO5	36.03957	32.60748	17.6776	3.026161	1.891988	1.785915	0.939365	0.934265	0.84247	0.744[illegible]
ENPO6	26.54116	16.26747	6.825924	5.812371	3.410853	3.407689	3.400306	2.961557	2.955229	2.134[illegible]
ENPO7	50.61988	16.80407	8.889756	5.352985	2.551258	1.351777	1.292621	1.230947	1.224654	1.214[illegible]
ENPO8	13.48388	9.855522	6.807562	6.657088	6.38216	6.343693	6.274678	4.636428	3.90442	3.666[illegible]
ENPR1	33.60939	29.08859	9.725811	9.486852	4.411539	4.090886	1.70845	0.9783	0.761808	0.588[illegible]
ENPR2	27.14176	8.44688	8.234206	8.170524	8.12126	6.25766	6.189172	3.224954	2.531661	1.939[illegible]
ENPR3	69.99692	1.796675	1.755188	1.591371	1.495633	1.329688	1.233951	1.220122	1.188209	1.187[illegible]
ENPR4	18.99112	9.769377	8.89804	8.416204	6.306316	3.30068	3.263534	2.979103	2.93665	2.585[illegible]
ENPR5	59.7242	10.74102	10.17594	5.096816	2.352122	1.705205	1.361289	1.094782	0.909	0.683[illegible]
ENPR6	35.89933	23.04615	9.47492	5.013873	3.80223	2.935583	2.902331	2.831669	2.080367	1.934[illegible]
ENPR7	51.95488	39.11536	3.669037	2.249064	0.872859	0.438534	0.244097	0.238205	0.121207	0.108[illegible]
ENPR8	20.11141	14.63539	10.94116	9.225893	8.251532	4.594197	3.784346	2.802122	2.202747	1.603[illegible]
GCPO1	26.96815	17.33463	15.60835	12.38014	4.449507	3.266321	3.025526	1.973998	1.673437	1.597[illegible]
GCPO2	33.77098	29.61213	27.27041	1.244703	1.008393	0.819956	0.770046	0.468546	0.424747	0.341[illegible]
